# Optimal Management Strategies for Primary HPV Testing for Cervical Screening: Cost-Effectiveness Evaluation for the National Cervical Screening Program in Australia

**DOI:** 10.1371/journal.pone.0163509

**Published:** 2017-01-17

**Authors:** Kate T. Simms, Michaela Hall, Megan A. Smith, Jie-Bin Lew, Suzanne Hughes, Susan Yuill, Ian Hammond, Marion Saville, Karen Canfell

**Affiliations:** 1 Cancer Research Division, Cancer Council NSW, Sydney, New South Wales, Australia; 2 Prince of Wales Clinical School, Faculty of Medicine, University of New South Wales, Sydney, New South Wales, Australia; 3 School of Public Health, Sydney Medical School, The University of Sydney, Sydney, New South Wales, Australia; 4 Steering Committee for the Renewal Implementation Project, National Cervical Screening Program, Department of Health, Canberra, Australian Capital Territory, Australia; 5 School of Women's and Infants' Health, University of Western Australia, Perth, Western Australia, Australia; 6 Victorian Cytology Service, Victoria, Australia; 7 Department of Obstetrics and Gynaecology, University of Melbourne, Melbourne, Victoria, Australia; Hospital Authority, CHINA

## Abstract

**Background:**

Several countries are implementing a transition to HPV testing for cervical screening in response to the introduction of HPV vaccination and evidence indicating that HPV screening is more effective than cytology. In Australia, a 2017 transition from 2-yearly conventional cytology in 18–20 to 69 years to 5-yearly primary HPV screening in 25 to 74 years will involve partial genotyping for HPV 16/18 with direct referral to colposcopy for this higher risk group. The objective of this study was to determine the optimal management of women positive for other high-risk HPV types (not 16/18) ('OHR HPV').

**Methods:**

We used a dynamic model of HPV transmission, vaccination, natural history and cervical screening to determine the optimal management of women positive for OHR HPV. We assumed cytology triage testing was used to inform management in this group and that those with high-grade cytology would be referred to colposcopy and those with negative cytology would receive 12-month surveillance. For those with OHR HPV and low-grade cytology (considered to be a single low-grade category in Australia incorporating ASC-US and LSIL), we evaluated (1) the 20-year risk of invasive cervical cancer assuming this group are referred for 12-month follow-up vs. colposcopy, and compared this to the risk in women with low-grade cytology under the current program (i.e. an accepted benchmark risk for 12-month follow-up in Australia); (2) the population-level impact of the whole program, assuming this group are referred to 12-month surveillance vs. colposcopy; and (3) the cost-effectiveness of immediate colposcopy compared to 12-month follow-up. Evaluation was performed both for HPV-unvaccinated cohorts and cohorts offered vaccination (coverage ~72%).

**Findings:**

The estimated 20-year risk of cervical cancer is ≤1.0% at all ages if this group are referred to colposcopy vs. ≤1.2% if followed-up in 12 months, both of which are lower than the ≤2.6% benchmark risk in women with low-grade cytology in the current program (who are returned for 12-month follow-up). At the population level, immediate colposcopy referral provides an incremental 1–3% reduction in cervical cancer incidence and mortality compared with 12-month follow-up, but this is in the context of a predicted 24–36% reduction associated with the new HPV screening program compared to the current cytology-based program. Furthermore, immediate colposcopy substantially increases the predicted number of colposcopies, with >650 additional colposcopies required to avert each additional case of cervical cancer compared to 12-month follow-up. Compared to 12-month follow-up, immediate colposcopy has an incremental cost-effectiveness ratio (ICER) of A$104,600/LYS (95%CrI:A$100,100–109,100) in unvaccinated women and A$117,100/LYS (95%CrI:A$112,300–122,000) in cohorts offered vaccination [Indicative willingness-to-pay threshold: A$50,000/LYS].

**Conclusions:**

In primary HPV screening programs, partial genotyping for HPV16/18 or high-grade triage cytology in OHR HPV positive women can be used to refer the highest risk group to colposcopy, but 12-month follow-up for women with OHR HPV and low-grade cytology is associated with a low risk of developing cervical cancer. Direct referral to colposcopy for this group would be associated with a substantial increase in colposcopy referrals and the associated harms, and is also cost-ineffective; thus, 12-month surveillance for women with OHR HPV and low-grade cytology provides the best balance between benefits, harms and cost-effectiveness.

## Introduction

Several countries are currently evaluating or implementing a transition from cytology to primary HPV testing for cervical screening[1,2,3], based on evidence indicating that HPV-based screening provides improved protection against invasive cervical cancer compared to cytology screening.[4] Furthermore, using the HPV test as a primary screening tool allows for development of population-based screening recommendations which take into account the impact of HPV vaccination, since management can be based on individual risk assessment at the time of screening (HPV infected versus uninfected), rather than on an individual’s HPV vaccination status, which may not be available at the point of screening.[5] Given HPV types 16/18 are associated with the greatest risk of developing CIN 3 or worse,[6,7,8] screening tests with partial genotyping for HPV 16/18 are expected to improve risk stratification of women who have a positive HPV test result in cervical screening programs.

Australia was the first country to initiate a national publicly-funded vaccination program in 2007. Three dose vaccination uptake is 72–73% in 12–13 year old females; catch-up in 18–26 year old females (conducted from 2007–9) achieved coverage rates of the order of 30–50%.[9,10] After the introduction of vaccination, Australia experienced rapid falls in vaccine-included HPV type infections, anogenital warts and histologically-confirmed cervical high-grade precancerous abnormalities (CIN 2/3). These have now been documented extensively in young females,[11,12,13,14,15,16] and reductions in infections have also been seen in unvaccinated females due to herd immunity.[15] Reductions have also been seen in anogenital warts[13,17] in both females and heterosexual males due to herd immunity effects, and rates of CIN2/3 have also decreased in women aged under 25 years and in women 25–29 years.[18] From 2013, males aged 12–13 were also included in the program, with a two-year catch-up for boys aged up to 15 years. Via herd immunity, male vaccination will also provide incremental benefits to females, and is expected to lead to further reductions in infections with vaccine-included types and high-grade cervical abnormalities in females. [5,19,20]

The implementation and rapid impact of HPV vaccination, together with accumulated evidence of primary HPV screening, promoted a review of the Australian National Cervical Screening Program (NCSP), known as the *Renewal*. This commenced in 2011, and led to an evaluation of the evidence in 2013, including modelled evidence of the impact of the renewed program in both unvaccinated cohorts and in cohorts offered vaccination, which was performed on behalf of the Australian Government’s Medical Services Advisory Committee (MSAC).[21,22] The MSAC evaluation identified several options for HPV screening in Australia that were predicted to result in improved outcomes, compared with the current practice of 2-yearly cytology-based screening. The greatest gains in effectiveness were associated with primary HPV testing with partial genotyping for HPV 16/18, in which women with these HPV types are referred directly for diagnostic colposcopic assessment. Based on the findings of the evaluation, in 2014, Australia announced an upcoming transition from the current 2-yearly conventional cytology-based program in women aged 18–20 to 69 years (‘pre-renewed NCSP’) to 5-yearly primary HPV-based screening with partial genotyping and direct referral for women testing HPV 16/18 in women aged 25–75 years (‘renewed NCSP’). The target date for implementation of the renewed NCSP is May 1^st^ 2017.

In the renewed NCSP, women testing HPV 16/18 positive will be referred directly to colposcopy (with a liquid-based cytology (LBC) sample taken to assist management at colposcopy), and HPV-negative women will be recalled at 5 years for routine screening, or discharged from screening (if aged 70 or older[23]). Women who test positive for high-risk HPV types (not 16/18) ('OHR HPV') will be triaged using LBC, with high-grade cytology referred to immediate colposcopy and normal cytology recalled for 12-month surveillance, but the optimal management of women with OHR HPV and low-grade cytology (ASC-US or LSIL) required evaluation. The current evaluation was undertaken as part of the process of developing detailed clinical management guidelines for the renewed cervical screening program.

Management of women who test positive for OHR HPV and have a liquid based cytology (LBC) test report of ASC-US or LSIL could potentially involve immediate colposcopy referral or a watch-and-wait approach of 12-month surveillance and re-testing for HPV at that time. Although some countries, in the context of HPV triage of low-grade cytology, currently recommend colposcopy referral for women with any high-risk HPV who have low-grade cytology,[24,25] it should be noted that because the higher risk HPV16/18 infections have already been removed from the pool of women being considered here, the remaining women are expected to be at lower risk than a group of women with low-grade cytology who are triage positive for any high-risk HPV type.

The potential impact of primary HPV screening programs has been evaluated in other settings. In Europe[26] and USA,[27] primary HPV screening (with cytology co-testing in the USA) was found to be cost-effective if offered to women aged over 30 years, however these studies did not consider partial genotyping for HPV types 16/18. Recently, three studies have evaluated the impact of a range of primary HPV screening strategies in England,[28] Australia[22] and New Zealand[29] and found that primary HPV screening with partial genotyping could be cost and life-years saving, although to date, there have been no specific modelled evaluations of the optimal management of women testing positive for high-risk HPV types other than 16/18. Other (non-modelling) studies have used clinical data to inform estimates of the potential sensitivity and specificity of different triage methods and referral thresholds,[30,31,32,33] however these have generally focussed on the cross-sectional performance, and not taken into account the full effectiveness of the strategy (including 12-month follow-up) or cost-effectiveness. Therefore, there is little direct evidence to inform the management of this group (which takes into account immediate versus longer term effects) and no randomised trials directly comparing these two alternative management strategies in this group. The aim of the current study was, therefore, in women positive for high-risk HPV (not 16/18) and low-grade cytology (ASC-US or LSIL), to estimate: (1) the 20-year risk of invasive cervical cancer if this group are referred for 12-month follow-up, and compare this to an accepted benchmark risk in Australia for 12-month follow-up: specifically, the risk for women with ASC-US/LSIL, who are currently followed-up at 12 months; (2) the population-level impact of the whole program, assuming this group are returned to 12-month follow-up vs. immediate colposcopy referral; and (3) the cost-effectiveness of immediate colposcopy compared to 12-month follow-up in this group. Evaluation was performed both for HPV-unvaccinated cohorts and for age-cohorts who were offered vaccination as adolescents, taking into account observed vaccination coverage.

## Methods

### Model platform

We used a comprehensive dynamic model of HPV transmission, vaccination, natural history and cervical screening to perform this evaluation. The platform has recently been used to perform the effectiveness modelling and economic evaluation of cervical screening, in unvaccinated cohorts and cohorts offered vaccination, for the application to MSAC for the NCSP renewal.[22] The platform has been further updated to incorporate management based on the draft Clinical Guidelines Recommendations for the Prevention of Cervical Cancer for the renewed National Cervical Screening Program, which were released for public consultation on 15^th^ February 2016.[23] The model has previously been used to evaluate the impact of the nonavalent HPV vaccine in Australia[34] as well as in England, New Zealand and USA.[35] The model has also been used to evaluate the cost-effectiveness of primary HPV screening in England[28] and New Zealand[29] and to evaluate changes to the cervical screening interval in Australia and the United Kingdom,[36,37] the role of alternative technologies for screening in Australia, New Zealand and England,[38,39,40,41] the role of HPV triage testing for women with low-grade cytology in Australia and New Zealand,[39,42] the role of HPV testing in the follow-up of women treated for precancerous lesions,[43] the cost-effectiveness of alternative screening strategies, and for combined screening and vaccination approaches in China.[44,45]

The model simulates HPV infection which can persist and/or progress to cervical intraepithelial neoplasia grades I, II and III (CIN1, CIN2, CIN3); CIN 3 can then progress to invasive cervical cancer. Progression and regression rates depend on the underlying HPV types present, and are different for HPV 16, HPV 18 and other high-risk HPV types. The model incorporates data on the age-specific risk of death due to causes other than cervical cancer,[46,47] rate of hysterectomy due to causes other than cervical cancer[48] and relative cervical cancer survival rates by extent of disease at diagnosis.[49]

The model has been calibrated to observed data for age-specific cervical cancer incidence and mortality, and the rate of histologically-confirmed high-grade squamous lesions per 1,000 women screened and after taking as input age-specific and recommendation-specific follow-up timing, it reproduces overall screening participation, as previously described.[22] Predictions from the dynamic HPV transmission and vaccination model have also recently been validated against observed declines in HPV prevalence in women aged 18–24 after the introduction of the quadrivalent HPV vaccine.[50]

### Cost and health-economic assumptions

We modelled a cohort of females from age 10 to 84 years. This cohort was selected in this evaluation on the basis that they would turn 18 years of age in 2017, and thus would be the first age group who would not be offered screening until age 25 if the pre-renewed NCSP were to change in that year. We took a health services perspective and considered aggregate costs for screening, diagnostic and treatment procedures in the year 2013 (detailed cost assumptions and methods described previously).[22] When discounting costs and effects, a discount rate of 5% was used for both costs and effects, in line with Australian recommendations.[51] Cost per life-year saved was considered as the primary outcome of the cost-effectiveness analysis, as we have previously found substantial variations in outcomes when alternate health utility weight sets (QALY weights) were considered.[22,28]

### Diagnostic test accuracy assumptions

Test accuracy for conventional cytology, HPV testing and liquid-based cytology are described in Table 1 and were calibrated to observed call rates (for conventional cytology) but were also consistent with findings from international meta-analyses for HPV test and liquid-based cytology.[22] In sensitivity analysis, we explored the potential for lower test accuracy rates, including the potential for HPV negative cancers. The test accuracy of colposcopy was based on a large colposcopy dataset (over 21,000 colposcopies) supplied by the Royal Women’s Hospital in Victoria.[41,42]

**Table 1:** Parameters used in the model and ranges explored in probabilistic sensitivity analysis*.

	Baseline values	Ranges explored in sensitivity analysis	Data informing assumptions
***Diagnostic test accuracy***	** **	** **	
Primary HPV test sensitivity (specificity) toCIN2+	96% (90%)	95% - 98% (87% - 93%)**for one-way sensitivity analysis, we assumed that in the worst case, the HPV test would be unable to detect the presence of 5.8% of cervical cancers (which are assumed to be truly HPV negative). [52]	Positivity rates informed by international meta-analyses.[53]
HPV test-of-cure sensitivity (specificity) toCIN2+	93% (81%)	86% - 97% (74% - 86%)	
Unsatisfactory rate for the HPV test	0%	0%-1%	
LBC test sensitivity (specificity) toCIN2+	ASC-US threshold: 77% (94%)	ASC-US threshold: 72% - 81% (92% - 95%)	Informed by a systematic review and meta-analysis comparing liquid-based and conventional cytology[54]
Unsatisfactory rate for LBC test	1.80%	0.3%-2.6%	Informed by data from a prospective Australia-based study[55]
Colposcopy sensitivity (specificity) to CIN2+	88% (52%)	80–91% (49–74%)	Informed by a large dataset of >20,000 colposcopies from the Royal Women’s Hospital in Victoria.[41,42]
Percent of positive HPV test results where HPV 16/18 is misclassified as other high-risk types	0%	0–5%	Assumption
Percent of positive HPV test results where OHR HPV types are misclassified as HPV 16/18	0%	0–5%	Assumption
***Screening attendance rates***		** **	
Probability of attending routine screening	Cumulative attendance in under 5 years is 9%; by 5 years is 80%; by 6 years in 85%	Attendance delayed by a year: cumulative attendance in under 6 years is 9%, and by 6 years is 80%.	Early and on-time attendance was informed by VCCR data and data from England (which has a call-recall system). Screening behaviour in under-screened women unchanged from current rates (based on VCCR data).
Probability of attendance for 12-month follow-up visits within a year	65–85% (depends on age)	Rate increased/decreased by 10%**	Informed by 12 month attendance as derived from VCCR data.
Probability of screening uptake at first invitation	82% at age 25, 91% attend before the age of 30	74% at age 25, 85% attend before the age of 30	Uptake at 25 years was assumed to be equivalent to update at or by 25 years as observed under current practice.
Probability of colposcopy attendance within a year after referral	85–95% (depends on age)	Rate increased/decreased by 10%**	Informed by data from the VCS and the Royal Women’s Hospital.[41,42]
***Cost assumptions***		** **	
cytology test cost	$30.50	$19.45-$42.00	Expert advice from the Renewal Steering committee
HPV test cost	$30.00	$20.00-$45.00**	
***Natural history assumptions***		** **	
Natural history aggressiveness		5% reduced progression rates; 5% increased progression rates^	This variation produces cancer rates that fit within uncertainty ranges derived from AIHW.[56]

* The uniform distribution is used for probabilistic sensitivity analysis when parameters from the ranges are selected for all parameters except for the choice of test sensitivity and specificity for the HPV and LBC test–in these cases, one of the three combinations of sensitivity and specificity are selected each time (i.e. there is no continuous distribution, just three discrete choices).

** Variations in these parameters were also explored in one-way sensitivity analysis.

### Compliance (adherence) assumptions

When modelling the pre-renewed NCSP, the model incorporated data on age-specific screening initiation and compliance with screening and management recommendations in Australian women informed by an analysis of data obtained from the Victoria Cervical Cytology Registry (VCCR). When modelling the renewed NCSP, we assumed a call-and-recall system would be introduced (based on MSAC recommendations);[57] that is, women would be sent invitations to attend for screening at age 25 years, and recall invitations when their next test was due (replacing the current system of reminders sent only when women are overdue for their next test). We assumed that the proportion of women who attend for their first screening test at age 25 years (the new initiation age) will be equivalent to the proportion who had their first screening test at or before the age of 25 under the pre-renewed NCSP. For the purposes of this evaluation, we assumed that no screening occurs before the age of 25 years under the renewed NCSP.

The behaviour of women re-attending after a negative test given the new call-and-recall system was informed by data from England, since a call-recall system has been implemented in that country. Specifically, the proportion of women who attended before or at the recommended screening interval (5-years under the renewed NCSP) was assumed to be similar to the proportion who attended at or before the recommended interval in England. However, we assumed that the coverage at 7 years or longer is equivalent to what is currently observed under the pre-renewed NCSP–i.e. that changing the recommended screening interval, by itself, will not change behaviour in women who have not attended screening in the past 7+ years.

As part of the initial MSAC evaluation of general screening approaches, we previously explored a range of other screening attendance assumptions, including lower screening uptake rates (reflecting a situation where no active invitation is sent at age 25) and a less ‘efficient’ call-recall system (in which there was a higher rate of early re-attendance and a lower rate of on-time attendance). The impact on these assumptions has been previously described.[22]

We assumed that the probability of attending a follow-up test in the renewed NCSP is equivalent to that currently observed under the pre-renewed NCSP for a given recall timeframe; that is, we assumed 65%-85% adherence to a recommendation to return for follow-up at 12 months (variation based on age), based on VCCR data. Compliance with colposcopy was informed by VCCR data as well as data from the Royal Women’s Hospital in Victoria (85–95%; variation based on age). We also considered the impact of varying colposcopy compliance by +/-10% and compliance with 12-month follow-up by +/-10% as part of the probabilistic sensitivity analysis (PSA) and one-way sensitivity analysis, for the cost-effectiveness outcome.

### Estimating the 20-year risk of invasive cervical cancer in women with high-risk HPV (not 16/18) and low-grade cytology

The guidelines for management of screen-detected abnormalities in the pre-renewed NCSP recommended that women who test ASC-US or LSIL at a routine cytology test should be referred for follow-up with another cytology test in 12 months.[58] An exception is made for women aged 30+ years who have a cytology result of ASC-US or LSIL and who do not have a history of negative cytology in the previous 2–3 years; these women are recommended to either return in 6 months or be referred directly to colposcopy. Therefore, the risk in women with LSIL (and who have a recent negative cytology in the last 2–3 years) who are subsequently followed-up in 12 months can be considered an acceptable risk benchmark in Australia for follow-up in 12 months. We estimated the risk of invasive cervical cancer over 20 years in this group and considered it as a benchmark when evaluating comparative risks in women testing OHR HPV and cytology ASC-US/LSIL.

We therefore evaluated the 20-year risk of developing invasive cervical cancer in women of different ages who attend a routine test under the renewed primary HPV screening program, and:

1. Tested OHR HPV and cytology ASC-US *or LSIL*, and returned in 12 months for an HPV test; women positive for any HPV type at 12 months were then immediately referred to colposcopy and HPV-negative women were returned to routine 5-yearly screening
2. Tested OHR HPV and cytology ASC-US, and returned in 12 months for an HPV test; management thereafter as specified above.
3. Tested OHR HPV and cytology *LSIL* and returned in 12 months for an HPV test; management thereafter as specified above.
4. Tested OHR HPV and cytology *ASC-US or LSIL at the year of the switch-over to primary HPV screening* and returned in 12 months for an HPV test; management thereafter as specified above, and
5. Tested OHR HPV and cytology *ASC-US or LSIL* and attended colposcopy.

In the switch-over scenario (Scenario iv), we assessed the risk of disease in women at the year of the transition from the pre-renewed NCSP to the renewed NCSP (2017) to examine potential transition effects, as the risk may be different in women who have previously been screened using cytology-based screening than in women who have been managed under the renewed NCSP over a lifetime.

In addition to the 20-year risk of cervical cancer, we also performed exploratory analysis to estimate the cumulative risk of CIN3+ at 24 months.

### Estimating the population-level impact

We evaluated the population-level impact of adopting a national program utilising primary HPV screening with partial genotyping, compared to current practice of two-yearly conventional cytology, under two alternative management options for women testing OHR HPV positive (not 16/18) and LBC ASC-US/LSIL:

1. 12-month follow-up with an HPV test; or
2. Immediate colposcopy.

For both options we estimated population-level cancer cases, cancer deaths, precancer treatments and colposcopy procedures. Case numbers were derived by applying predicted age-specific rates to the projected Australian female population for 2017. These predictions did not consider transitional impacts, but were based on long term outcomes.

### Estimating the cost-effectiveness of referring women who test positive for high-risk HPV (not 16/18) and LBC ASC-US/LSIL to colposcopy compared to 12-month follow-up

We estimated the incremental cost-effectiveness ratios (ICERs) for immediate colposcopy referral versus 12-month follow-up for women who tested positive for OHR HPV and cytology ASC-US/LSIL, using standard methods. In the main evaluation we assumed the referral decision applied for women of all ages, but because the relative proportion of high-grade abnormalities attributed to OHR HPV (vs. HPV16/18) increases in older women,[59,60,61,62] it is possible that immediate colposcopy may be more cost-effective in older women. We therefore also assessed strategies which utilised 12-month follow-up for younger women, but recommended immediate colposcopy for women over the age of either 35, 45, 55 or 65 years.

### Vaccination assumptions

The population-level outcomes and the cost-effectiveness outcomes were evaluated separately for unvaccinated cohorts and for cohorts offered vaccination at age 12 years in 2011, with uptake informed by the National HPV Vaccination Register (~72% coverage)[9]. Vaccine uptake was also modelled in other cohorts of females and in boys (included in the program since 2013), based on published data[9] in order to capture indirect protection.

### Sensitivity analysis

Probabilistic sensitivity analysis was performed on the cost-effectiveness outcome to assess the impact of uncertainties in screening attendance rates, screening test accuracy, natural history parameters, and costs. The ranges of variation in these parameters are shown in shown in Table 1, and details of the choice of ranges has been previously described.[22]

One-way sensitivity analysis was also performed to isolate the impact of key parameters, including compliance with 12-month follow-up, compliance with colposcopy referral, HPV test accuracy rate (including lower positivity rate of the HPV test to cervical cancer to capture the potential effect of a proportion of HPV negative cancers), colposcopy accuracy rates and the HPV test cost.

### Supplementary analysis: management options after 12-month surveillance with HPV testing

As a further exploratory analysis, we evaluated the population-level impact of adopting a national cervical screening program utilising primary HPV screening with partial genotyping, assuming women testing OHR HPV positive (not 16/18) and LBC ASC-US/LSIL are referred for follow-up in 12 months with an HPV test, with two possible management options at 12 months:

1. Women who test negative for high-risk HPV type at 12 months are returned to routine 5-yearly screening; women who test positive for any high-risk HPV type are referred for colposcopy (“12-month follow-up”);
2. Women who test negative for high-risk HPV type at 12 months are recommended to return for another follow-up HPV test 12 months later (i.e. at 24 months). Women who test HPV-negative at both follow-up tests are returned to routine 5-yearly screening. Women who test positive for any high-risk HPV type at either follow-up test (12 or 24 months) are referred for colposcopy (“12- and 24-month follow-up”).

We evaluated long term population-level outcomes for cancer cases, deaths, precancer treatments and colposcopy procedures, as well as the cost-effectiveness of 12- and 24-month follow-up compared to 12-month follow-up only for both unvaccinated cohorts and cohorts offered vaccination. These predictions for exploratory analysis did not consider transitional impacts.

## Results

### 20-year risk of cervical cancer in women testing positive for high-risk HPV (not 16/18) and cytology low-grade

**Fig 1:**
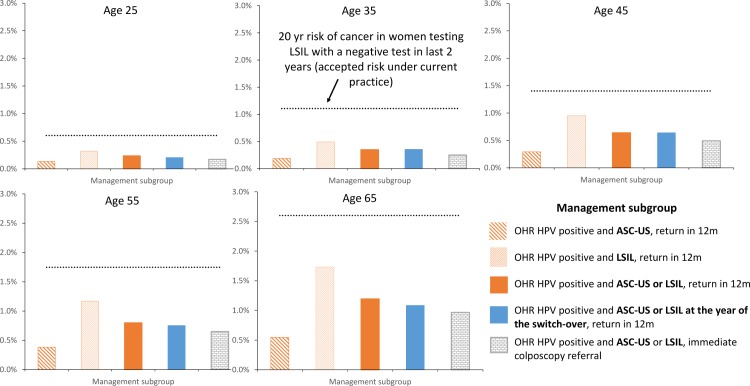
The cumulative risk of developing invasive cervical cancer after 20 years* by age and management subgroup. The 20-year risk of cervical cancer in women with LSIL in the pre-renewed NCSP with a negative test in last 2 years (accepted risk for 12-month repeat recommendation under the pre-renewed NCSP) is shown as the horizontal line in each graph. *The risk was evaluated in unvaccinated cohorts. It was assumed that the risk in cohorts offered vaccination would be equal to or lower than in unvaccinated cohorts (due to the potential impact of vaccine cross-protection against OHR HPV types).

The 20-year risk of cancer in women who test positive for OHR HPV and cytology low-grade who are aged 25, 35, 45, 55 and 65 years, considering various management strategies, is shown in Fig 1. The benchmark risk in women testing LSIL with a negative test result in the last two years under the pre-renewed NCSP is also shown. The 20-year risk of cervical cancer in women who tested positive for OHR HPV and cytology ASC-US/LSIL, and who are referred to colposcopy (≤1.0% at all ages), is slightly lower than in the group who underwent follow-up at 12 months (≤1.2% at all ages), both of which are lower than the estimated ≤2.6% benchmark risk in women testing LSIL with a negative test result in the last two years under the pre-renewed NCSP (accepted risk for 12-month repeat recommendation under the pre-renewed NCSP). We also found that the risk in women who test positive for OHR HPV and cytology ASC-US/LSIL at the time of the transition was similar to the new primary HPV screening program as it was for cohorts always screened with primary HPV testing.

**Fig 2:**
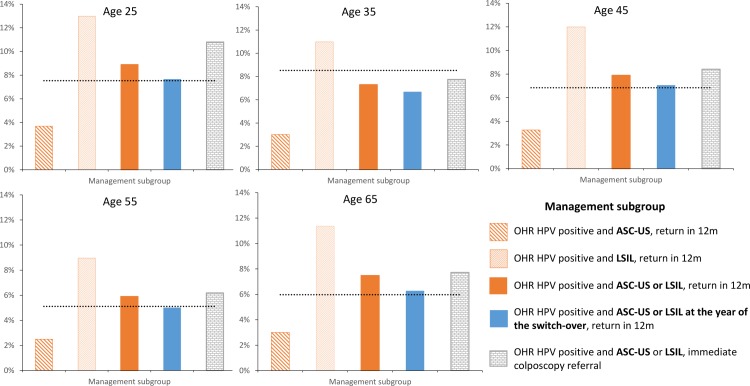
The cumulative risk of CIN3+ at 24 months* by age and management subgroup. The 24-month risk of CIN3+ in women with LSIL with a negative test in last 2 years (accepted risk for 12-month repeat recommendation under the pre-renewed NCSP) is shown as the horizontal line in each graph. *The risk was evaluated in unvaccinated cohorts. It was assumed that the risk in cohorts offered vaccination would be equal to or lower than in unvaccinated cohorts (due to potential the impact of vaccine cross-protection against OHR HPV types).

Further exploratory analysis was done to investigate the cumulative risk of CIN3+ at 24 months after the test result, and compare this to the cumulative risk of CIN3+ at 24 months in women testing LSIL with a negative test result in the last two years under the pre-renewed NCSP (shown in Fig 2). For all ages, the 24-month risk of CIN3+ in women who tested positive for OHR HPV and cytology ASC-US/LSIL under the renewed NCSP and who were referred to colposcopy was higher than in the group who underwent follow-up at 12 months. Furthermore, for some ages, the risk of CIN3+ was higher in women testing positive for OHR HPV and cytology ASC-US/LSIL under the renewed NCSP (regardless of whether they were referred to colposcopy or for 12-month follow-up) when compared to the current benchmark. The additional CIN3+ cases under the renewed NCSP are due to the more sensitive HPV test detecting more CIN3 in women testing positive for OHR HPV and cytology ASC-US/LSIL than is being detected in women testing LSIL with a negative test result in the last two years under the pre-renewed NCSP (accepted risk for 12-month repeat recommendation under the pre-renewed NCSP).

### Population-level impact

**Fig 3:**
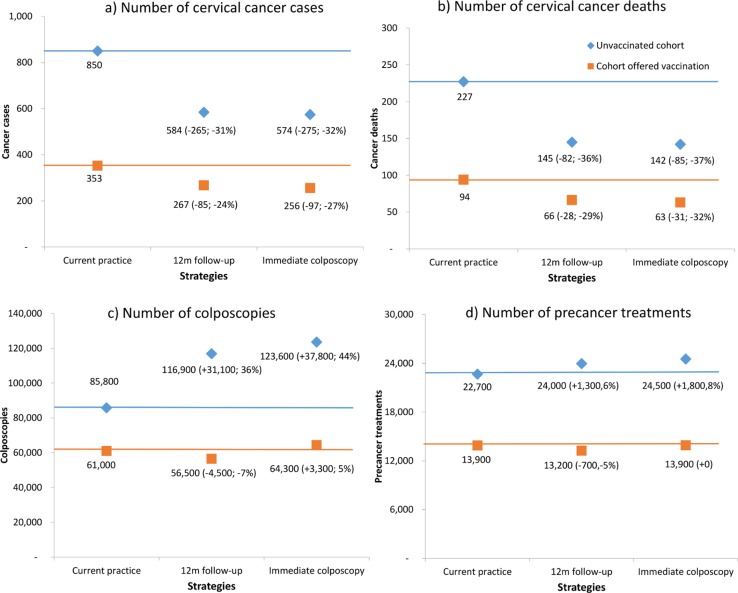
Model predicted annual number* of cancer cases (a), cancer deaths (b), colposcopies(c) and precancer treatments d) for unvaccinated cohorts (diamond) and cohorts offered vaccination (squares) under three strategies–pre-renewed NCSP, primary HPV screening in which women testing OHR HPV and ASC-US/LSIL are referred for 12-month follow-up, and primary HPV screening in which this group is referred for immediate colposcopy.** *Case numbers derived by applying predicted age-specific rates to the projected Australian female population for 2017.[63] **Numbers in parentheses represent the difference in case numbers and percentage difference compared to the pre-renewed NCSP.

Fig 3 shows the model-predicted number of cervical cancer cases, deaths, histologically-confirmed high-grade lesions and colposcopies for unvaccinated cohorts and cohorts offered vaccination. Results are shown for the pre-renewed NCSP and for the renewed NCSP, assuming either 12-month follow-up for women who test positive for OHR HPV and cytology ASC-US/LSIL, or immediate colposcopy referral, and use ABS data for population projections in 2017.[63]

In the absence of HPV vaccination under the renewed NCSP, compared with the pre-renewed NCSP, cancer cases would be predicted to drop by 31% (265 fewer cases) or 32% (275 fewer cases) assuming either 12-month follow-up or immediate colposcopy for women with OHR HPV and ASC-US/LSIL, respectively, and to drop by 24% (85 less cases) or 27% (97 less cases) respectively in cohorts offered vaccination. For unvaccinated cohorts, cancer deaths are predicted to drop by 36% (82 less deaths) and 37% (85 less deaths) under 12-month follow-up and immediate colposcopy options respectively, and drop by 29% (28 less deaths) and 32% (31 less deaths) in cohorts offered vaccination. Thus, overall, immediate colposcopy provides an additional 1–3 percentage point reduction in cervical cancer cases and deaths when the findings of the whole program are considered at the population level in relation to current screening practice in the pre-renewed NCSP.

For unvaccinated cohorts, colposcopies are predicted to increase substantially under the renewed NCSP compared to the pre-renewed NCSP: by 36% (31,100 additional colposcopies) given 12-month follow-up or 44% (37,800 additional colposcopies) given immediate colposcopy. In cohorts offered vaccination, colposcopies are expected to *decrease* by 7% (4,500 fewer colposcopies) given the 12-month follow-up option compared to the pre-renewed NCSP, or increase by 5% (3,300 additional colposcopies) given the immediate colposcopy option. Thus, immediate colposcopy results in an *additional* 8% (6,700) colposcopies over 12-month follow-up in unvaccinated cohorts when compared to the pre-renewed NCSP, and an additional 12% (7,800) colposcopies in cohorts offered vaccination.

Similarly, the number of treatments for CIN2/3 is predicted to increase in unvaccinated cohorts by 6% and 8% under 12-month follow-up and immediate colposcopy options, respectively, but to *decrease* in cohorts offered vaccination by 5% under 12-month follow-up and remain unchanged under the immediate colposcopy option.

Table 2 shows the predicted number of cervical cancer cases, deaths, colposcopies and precancer treatments given the alternative management options, as well as the number of additional colposcopies and additional precancer treatments required to avert a cancer case or death under the immediate colposcopy option, compared to 12-month follow-up. Although immediate colposcopy referral provides some incremental benefit over 12-month follow-up, this comes at the cost of a marked increase in the number of colposcopies, and also an increase in the number of precancer treatments. An additional 653–684 colposcopies and 55–59 precancer treatments would be required to avert a cervical cancer case given immediate colposcopy compared to 12-month follow-up, and an additional 2,337–2,452 colposcopies and 195–211 precancer treatments would be required to avert each cancer death for both unvaccinated cohorts and for cohorts offered vaccination. If 12-month follow-up was recommended for younger women and immediate colposcopy recommended only at older ages, an additional 340–451 colposcopies and 12–22 precancer treatments would be required to avert each cancer case (or 888–1,487 colposcopies and 32–74 precancer treatments required to avert each cancer death)—for example, if only women over age 45 years are referred to colposcopy, the number of additional colposcopies per case averted still exceeds 400.

**Table 2:** Numbers* of cervical cancer cases, deaths and colposcopies predicted in a year under a range of screening scenarios for unvaccinated cohorts and cohorts offered vaccination at age 12 years**.

**Unvaccinated cohorts**						
	# cancer cases	# cancer deaths	# precancer treatments	# colposcopies	# colposcopies required to avert a cancer case (# required to avert a cancer death) compared to 12-month follow-up	# precancer treatments required to avert a cancer case (# required to avert a cancer death) compared to 12-month follow-up
Pre-renewed NCSP (current practice)	850	227	22,700	85,800	-	-
12m follow-up	584 (-265; -31%)	145 (-82;-36%)	24,000 (+1,300; 6%)	116,900 (+31,100; 36%)	-	-
Immediate colposcopy	574 (-275; -32%)	142 (-85; -37%)	24,500 (+1,800; 8%)	123,600 (+37,800; 44%)	653 (2337)	55 (195)
12m follow-up for women less than age 35, Immediate colposcopy if aged 35+	577 (-272; -32%)	143 (-84; -37%)	24,100 (+1,400; 6%)	120,100 (+34,300; 40%)	451 (1487)	22 (73)
12m follow-up for women less than age 45, immediate colposcopy if aged 45+	581 (-269; -32%)	144 (-84; -37%)	24,000 (+1,300; 6%)	118,500 (+32,700; 38%)	414 (1195)	14 (41)
12m follow-up for women less than age 55, immediate colposcopy if aged 55+	583 (-267; -31%)	144 (-83; -37%)	24,000 (+1,300; 6%)	117,700 (+31,900; 37%)	401 (1066)	12 (32)
12m follow-up for women less than age 65, immediate colposcopy if aged 65+	584 (-266; -31%)	145 (-82; -36%)	24,000 (+1,300; 6%)	117,100 (+31,300; 36%)	341 (901)	12 (32)
**Cohorts offered vaccination at age 12 years**						
	# cancer cases	# cancer deaths	# precancer treatments	# colposcopies	# colposcopies required to avert a cancer case (# required to avert a cancer death) compared to 12 month follow-up	# precancer treatments required to avert a cancer case (# required to avert a cancer death) compared to 12 month follow-up
Pre-renewed NCSP (current practice)	353	94	13,900	61,000	-	-
12m follow-up	267 (-85; -24%)	66 (-28; -29%)	13,200 (-700; -5%)	56,500 (4,500; -7%)	-	-
Immediate colposcopy	256 (-97; -27%)	63 (-31; -33%)	13,900 (0; 0%)	64,300 (3,300;5%)	684 (2452)	59 (211)
12m follow-up for women less than age 35, Immediate colposcopy if aged 35+	259 (-93; -26%)	64 (-30; -32%)	13,400 (-500; -3%)	60000 (-1,000; -2%)	451 (1482)	22 (74)
12m follow-up for women less than age 45, immediate colposcopy if aged 45+	263 (-90; -25%)	65 (-29; -31%)	13,300 (-600; -4%)	58200 (-2,800; -5%)	413 (1191)	14 (41)
12m follow-up for women less than age 55, immediate colposcopy if aged 55+	265 (-88; -25%)	65 (-28; -30%)	13,300 (-600; -5%)	57400 (-3,600; -6%)	399 (1059)	12 (32)
12m follow-up for women less than age 65, immediate colposcopy if aged 65+	267 (-86; -24%)	66 (-28; -30%)	13,200 (-700; -5%)	56700 (-4,300; -7%)	340 (888)	12 (32)

*Case numbers derived by applying predicted age-specific rates to the projected Australian female population for 2017.[63].

**Numbers in parentheses represent. the difference in case numbers and percentage difference compared to the pre-renewed NCSP.

### The cost-effectiveness of referring women testing positive for high-risk HPV (not 16/18) and LBC ASC-US/LSIL to colposcopy compared to 12-month follow-up

Table 3 shows the cost-effectiveness of implementing immediate colposcopy referral for women testing positive for OHR HPV and cytology ASC-US/LSIL in the renewed NCSP, compared to 12-month follow-up for this group. We found that immediate colposcopy had an ICER of $104,600/LYS (95%CrI:$100,100–109,100) in unvaccinated women and an ICER of $117,100/LYS (95%CrI:$112,300–122,000) in cohorts offered vaccination, compared to 12-month follow-up (and in the context of an indicative willingness-to-pay threshold of A$50,000/LYS in Australia).

**Table 3:** The cost-effectiveness of immediate colposcopy compared to 12-month follow-up in women with OHR HPV and cytology ASC-US or LSIL.

	Incremental cost-effectiveness of immediate colposcopy compared to 12m follow-up in women with OHR HPV and cytology ASC-US or LSIL (A$/LYS)	
	Unvaccinated cohorts	Cohorts offered vaccination
Immediate colposcopy for women of all ages*	$104,600/LYS (95%CrI:$100,100–109,100)	$117,100/LYS (95%CrI:$112,300–122,000)
**12m follow-up for women aged less than 35, then Immediate colposcopy for women aged 35+**	$59,800/LYS (95%CrI:$55,800-$62,100)	$61,600/LYS (95%CrI:$58,300-$64,900)
**12m follow-up for women aged less than 45, then immediate colposcopy for women aged 45+**	$39,800/LYS (95%CrI:$36,700-$41,900)	$40,900/LYS (95%CrI:$38,300-$43,600)
**12m follow-up for women aged less than 55, then immediate colposcopy for women aged 55+**	$40,100/LYS (95%CrI:$36,100-$41,500)	$40,200/LYS (95%CrI:$37,500-$42,900)
**12m follow-up for women aged less than 65, then immediate colposcopy for women aged 65+**	$38,100/LYS (95%CrI:$35,000-$40,500)	$39,400/LYS (95%CrI:$36,600-$42,200)

LYS = life-years saved.

*The ICER for immediate colposcopy for women of all ages was calculated against 12-month follow-up for women of all ages. If the ICER was instead calculated compared to immediate colposcopy for women aged 35+ years (which was the next most effective strategy), then the calculated ICER increased and was >$140,000/LYS for both unvaccinated cohorts and cohort offered vaccination.

The ICERs were more favourable if immediate colposcopy referral was only recommended for older women. If immediate colposcopy was recommended for women aged 35 years and older, the ICER remained above A$50,000/LYS, but recommending immediate colposcopy for women aged 45 and older reduced the ICER to A$39,800 (95%CrI: A$36,700-A$41,900) in unvaccinated cohorts and A$40,900/LYS (95%CrI: A$38,300-A$43,600) in cohorts offered vaccination. Therefore, it may be considered cost-effective to utilise immediate colposcopy for women aged 45 and older.

### One-way sensitivity analysis

**Fig 4:**
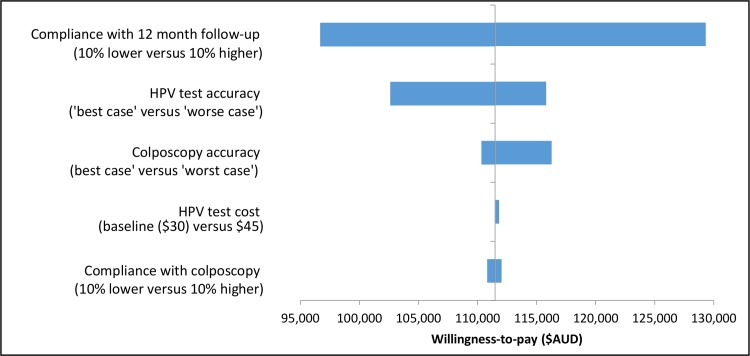
The incremental cost-effectiveness ratio (ICER) of immediate colposcopy compared to 12-month follow-up in women with OHR HPV and ASC-US/LSIL for parameters included in one-way sensitivity analysis–unvaccinated cohorts.

**Fig 5:**
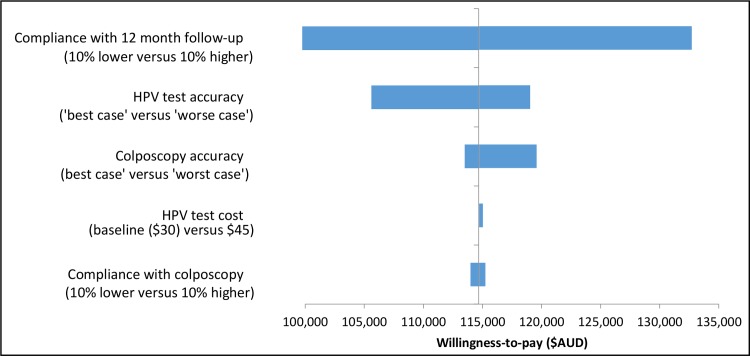
The incremental cost-effectiveness ratio (ICER) of immediate colposcopy compared to 12-month follow-up in women with OHR HPV and ASC-US or LSIL for parameters included in one-way sensitivity analysis–cohorts offered vaccination.

One-way sensitivity analysis was also performed to isolate the impact of key assumptions, including compliance rates with 12-month follow-up, compliance with colposcopy referral, HPV test accuracy rate, HPV test cost and colposcopy accuracy rates on the predicted cost-effectiveness of immediate colposcopy referral vs. 12-month follow-up. The results are shown as tornado diagrams in Fig 4 for unvaccinated cohorts and Fig 5 for cohorts offered vaccination. The cost-effectiveness of immediate colposcopy referral vs. 12-month follow-up was most sensitive to the assumptions around compliance with 12 month follow-up, followed by HPV test accuracy assumptions. However, even considering uncertainty in these parameters, it still remained cost-ineffective to refer women who test positive for OHR HPV types and cytology ASC-US/LSIL in the renewed NCSP in Australia (ICER>$95,000/LYS).

### Supplementary analysis: Follow-up options after 12-month surveillance with HPV testing

**Fig 6:**
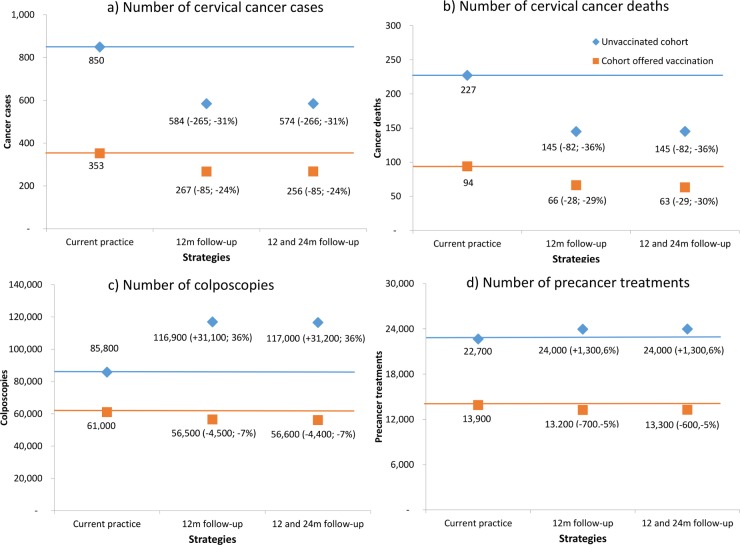
Model predicted annual number* of cancer cases (a), cancer deaths (b), colposcopies (c) and precancer treatments (d) for unvaccinated cohorts (diamond) and cohorts offered vaccination (squares) under three strategies–current practice, primary HPV screening in which women testing OHR HPV and ASC-US/LSIL are referred for 12-month follow-up, and primary HPV screening in which this group is referred for 12- and 24-month follow-up.** *Case numbers derived by applying predicted age-specific rates to the projected Australian female population for 2017.[63]** Numbers in parentheses represent the difference in case numbers and percentage difference compared to the pre-renewed NCSP.

Fig 6 shows the model-predicted number of cervical cancer cases, deaths, colposcopies and precancer treatments for unvaccinated cohorts and cohorts offered vaccination, depending on whether women with OHR HPV and ASC-US/LSIL cytology require only one negative HPV test (at 12 months) before being returned to routine screening, or two consecutive negative HPV tests (at 12 months and 24 months).

For both unvaccinated cohorts and cohorts offered vaccination, the population-level number of cervical cancer cases and deaths was very similar between the 12-month follow-up scenario and the 12- and 24-month follow-up scenarios (<1% difference). The number of precancer treatments and the number of colposcopies predicted under the two scenarios was also very similar, and there was at most 1% difference between the two scenarios in terms of these outcomes.

The ICER for 12- and 24-month follow-up was >A$200,000/LYS compared with 12-month follow-up alone (Table 4). Even if the switch to repeat 12- and 24-month follow-up is confined to women over 55 years, the ICER remains greater than A$70,000/LYS. Therefore, repeat 12- and 24-month follow-up is unlikely to be cost-effective in Australia, compared to 12-month follow-up alone.

**Table 4:** The cost-effectiveness of 12- and 24-month HPV testing, compared to 12-month HPV testing only, in women with OHR HPV and cytology ASC-US/LSIL.

	Incremental cost-effectiveness ratio (ICER) of 12- and 24-month follow-up compared to 12-month follow-up only (A$/ LYS)	
	Unvaccinated cohorts	Cohorts offered vaccination
12 and 24m follow-up for all ages*	$ 238,000	$228,000
**12m follow-up for women aged less than 35, then 12 and 24m follow-up for women aged 35+**	$147,000	$143,000
**12m follow-up for women aged less than 45, then 12 and 24m follow-up for women aged 45+**	$97,000	$ 94,000
**12m follow-up for women aged less than 55, then 12 and 24m follow-up for women aged 55+**	$72,000	$71,000
**12m follow-up for women aged less than 65, then 12 and 24m follow-up for women aged 65+**	Extended dominated	Extended dominated

LYS = life-years saved.

*The ICER for immediate colposcopy for all ages was calculated compared to 12-month follow-up for all ages. If the ICER was instead compared to immediate colposcopy for women aged 35+ years (which was the next most effective strategy), then the calculated ICER increased, and was >$300,000/LYS for both unvaccinated cohorts and cohort offered vaccination.

Extended dominated = the strategy has a higher ICER than a more effective strategy.

## Discussion

In this evaluation of potential management strategies for women who test positive for high-risk HPV (not 16/18) and cytology ASC-US/LSIL in the renewed NCSP in Australia, we found that recommending 12-month follow-up surveillance with HPV testing resulted in a lower 20-year risk of developing invasive cervical cancer than the current risk in women with a screening cytology result of LSIL (who have also had a negative cytology result within the last 2 years) in the pre-renewed cytology-based screening program. This indicates that the long-term risk of invasive cancer in the group testing positive for high-risk HPV (not 16/18) and cytology ASC-US/LSIL is lower than an accepted benchmark risk for 12-month follow-up in Australia in the current cervical screening program. We note that this is a conservative benchmark, as data from safety monitoring reports indicate that women testing LSIL under the current program without a negative cytology in the last 2 years are often referred to 12-month follow-up in Australia, and not to 6-month follow-up or colposcopy, as recommended by the guidelines.[64] We also found that the incremental benefit of direct referral to colposcopy, in terms of overall population impact on invasive cancer incidence and mortality, while positive, was relatively modest and that, consequently, the management of these women via direct referral to colposcopy is unlikely to be cost-effective. We also found that such a management strategy would be associated with some harms in the form of additional colposcopy referrals and treatments, with over 650 additional colposcopies required to avert each additional case of cervical cancer and over 2,300 additional colposcopies to avert a cancer death in both unvaccinated cohorts and cohorts offered vaccination, although we note that these predictions are predicated on the overall screening adherence assumptions used for the evaluation.

These findings are reflective of the lower risks seen in the group of women testing positive for high-risk HPV (not 16/18) and cytology ASC-US/LSIL, once the higher risk groups (those positive for HPV16/18 infection or cytology results suggestive of high-grade, who are referred to colposcopy), are removed from the population of interest. In the renewed NCSP, women who test positive for HPV16/18 and/or OHR HPV with high-grade cytology are already managed as ‘higher risk’ and directly referred to colposcopy. It is likely that in many cases, the low-grade cytology is reflective of a productive HPV infection with the OHR HPV type. By managing this group using a follow-up surveillance HPV test, it is possible to check for persistent HPV infection, which has been shown to increase the risk of subsequent disease progression.[65,66] At the 12-month follow-up visit, we assumed that women with any HPV detected would at that point be referred for colposcopy. In this way only women with transient OHR infections are not referred to colposcopy; and our findings indicate that this has only a very a modest impact on the risk of developing invasive cervical cancer in the future.

We also found that direct referral of women testing positive for high-risk HPV (not 16/18) and cytology ASC-US/LSIL would be more cost-effective if such referral is confined to older women (45 years or older); the cost-effectiveness of referring older women in this group direct to colposcopy would need to be balanced against any difficulties inherent in implementing differential management recommendations in a specific subgroup according to the age of the woman at testing, and is a policy decision which would need to take into account a range of other factors, such as the number of colposcopies required to avert each additional case, which still exceeds 400 for women aged 45 or older.

In the context of performing 12-month follow-up with an HPV test, adding a second HPV test at 24 months for those who were negative at the 12-month visit would be associated with very marginal benefits and would be very cost-ineffective, with an incremental cost-effectiveness ratio of >$200,000/LYS, compared to 12-month follow-up only in this group: this intervention remains cost-ineffective even if it confined only to older women. This finding likely reflects the predicted effectiveness of a single HPV test to check for persisting infection at 12 months.

Our findings are relevant for unvaccinated cohorts and cohorts offered the quadrivalent vaccine; however, cervical screening in the future will require re-evaluation for cohorts offered the nonavalent vaccine. A recent study found that only a few cervical screens per lifetime would remain cost-effective for these cohorts in Australia.[35] However, cohorts offered the nonavalent vaccine at ages 12–13 years would not start cervical screening until age 25, and so it would still be at least another 13 years until cervical screening would need to be re-evaluated due to younger cohorts offered the nonavalent vaccine entering the screening program.

Our evaluation has several strengths. Firstly, we have used a comprehensive and well-validated model of cervical cancer natural history, HPV infection, and cervical screening, which has been validated against many data sources across several countries.[22,28,36,37,38,39,40,41,43,44,45] By necessity, we were required to make assumptions about compliance with 12-month follow-up and colposcopy under the renewed NCSP; however, these rates were assumed to be equivalent to the observed rates in women referred for 12-month follow-up or colposcopy under the pre-renewed NCSP, which may be conservative given the planned change from a reminder-based to an active call-recall invitation system. We also assumed screening participation in cohorts offered vaccination would not change from current observed rates. Recent evidence suggests lower screening participation in younger women who have been vaccinated; however, we also explored varying compliance with follow-up and colposcopy attendance rates as part of probabilistic sensitivity analysis and one-way sensitivity analysis for the cost-effectiveness outcome.[67] Our modelling took a whole-of-cohort approach, and therefore did not consider different screening participation rates in sub-populations such as migrant or Indigenous women, in which screening participation has been reported to be lower than the general population.[68,69] Special care will need to be taken for women in these higher risk groups referred for either colposcopy or for 12-month follow-up to ensure that these women are not lost to follow-up.

In our evaluation, we assumed that the HPV test technologies would provide information separately for HPV types 16/18, but that other high-risk HPV types would be managed in the same way as each other. However, there is evidence to suggest that there is heterogeneity in the risk in women positive for types other than 16/18 –for instance, the risk of high-grade CIN in women positive for HPV types 33 and 31 is significantly higher than some of the other high-risk HPV types,[70] and more aggressive management of women who test positive for these types could be justified. Genotyping for types 16/18 was chosen in Australia because they are known to be the highest risk types, it allows for the potential for surveillance of vaccine impact, and the resulting impact on resource utilization (such as colposcopy referrals) will be minimized by the high vaccine uptake in younger women, as has been shown previously.[22] Our recent work has also shown that there will be fluctuations in test procedures after the transition to longer-interval HPV testing, including a predicted ~50% increase in colposcopies after the first round of screening, even taking into account the impact of HPV vaccination in Australia.[71] Therefore, more aggressive management of other high-risk HPV types (such as 31/33), when implemented with a screening start age of 25 years (as opposed to starting screening at older ages), will place further demands on the health system during the transition. Furthermore, the cost-effectiveness of colposcopy referral for other non-16/18 types compared with other triage options for this group (such as the potential use of dual-stained cytology with p16/Ki67) has not yet been demonstrated. Finally, it is notable that an ongoing major clinical trial, *Compass* [Clinicaltrials.gov NCT02328872], is expected to provide direct evidence in the future on the outcomes in women with OHR HPV and ASC-US/LSIL cytology within a primary HPV screening program. Compass is a large scale randomised controlled trial of 5-yearly HPV versus 2.5 yearly image read LBC cytology screening in women aged 25–69 years, and it is being conducted in the state of Victoria, Australia. HPV screening in the trial incorporates the use of partial genotyping and 12-month follow-up for women testing positive for high-risk HPV (not 16/18) and cytology ASC-US/LSIL, as considered in the current evaluation. Recruitment is stratified according to whether women are in age cohorts that were offered vaccination. In HPV-screened women, a secondary randomisation process for women testing positive for other high-risk HPV types (i.e. not HPV16/18) is implemented, and these women are randomised to be triaged either with LBC or with dual-stained p16/Ki67 cytology (CINtec PLUS, Roche/Ventana). In conjunction with the implementation of the renewed NCSP, Compass will provide emergent evidence both on the performance of LBC triage of women with OHR HPV infections, but also data on new options for management in this group. In the future, this is expected to provide the basis for further review and, if warranted, consideration of the role of other options for management.

To our knowledge, this is the first detailed evaluation of the management of women testing positive for high-risk HPV (not 16/18) in a primary HPV screening program. These findings are currently informing the development of new clinical management guidelines in Australia[23] and will be of broad interest for other countries introducing primary HPV screening, especially in the context of considering the overall benefits of partial genotyping options for primary screening. Although currently there is an absence of direct clinical evidence to support our modelled findings, it should be noted that these findings are broadly consistent with those from a systematic review on the longitudinal risks of serious disease in women positive for high-risk HPV (not 16/18) and cytology LSIL.[23] Our benchmark for the acceptable risk for 12-month follow-up may not be directly applicable in other settings; however, Australia has one of the lowest rates of cervical cancer worldwide and recommends more frequent routine screening than many settings, and so this benchmark risk may be lower than what is accepted in other settings and should thus be considered conservative.

We conclude that 12-month surveillance of women who test positive for other high-risk HPV (not 16/18) and cytology ASC-US/LSIL, appears to provide the best balance of benefits, harms and cost-effectiveness in the context of partial genotyping for HPV 16/18 within the new Australian primary-HPV based screening program.
